# Body composition dynamics and impact on clinical outcome in gastric and gastro-esophageal junction cancer patients undergoing perioperative chemotherapy with the FLOT protocol

**DOI:** 10.1007/s00432-022-04096-w

**Published:** 2022-07-21

**Authors:** Florian Huemer, Stefan Hecht, Bernhard Scharinger, Verena Schlintl, Gabriel Rinnerthaler, Konstantin Schlick, Ronald Heregger, Thomas Melchardt, Angela Wimmer, Iris Mühlbacher, Oliver Owen Koch, Daniel Neureiter, Eckhard Klieser, Sara Seyedinia, Mohsen Beheshti, Richard Greil, Lukas Weiss

**Affiliations:** 1 Department of Internal Medicine III with Haematology, Medical Oncology, Haemostaseology, Infectiology and Rheumatology, Oncologic Center, Salzburg Cancer Research Institute , Center for Clinical Cancer and Immunology Trials (SCRI-CCCIT), Paracelsus Medical University Salzburg, Salzburg, Austria; 2 Cancer Cluster Salzburg, Salzburg, Austria; 3 Department of Radiology, Paracelsus Medical University Salzburg, Salzburg, Austria; 4 Department of Surgery, Paracelsus Medical University Salzburg, Salzburg, Austria; 5 Institute of Pathology, Paracelsus Medical University Salzburg, Salzburg, Austria; 6 Division of Molecular Imaging and Theranostics, Department of Nuclear Medicine, Paracelsus Medical University Salzburg, Salzburg, Austria

**Keywords:** Gastric cancer, Perioperative chemotherapy, Sarcopenia, Skeletal muscle index, Body mass index

## Abstract

**Purpose:**

Perioperative chemotherapy with FLOT constitutes a standard of care approach for locally advanced, resectable gastric or gastro-esophageal junction (GEJ) cancer. We aimed at investigating anthropometric, CT-based and FDG-PET-based body composition parameters and dynamics during this multidisciplinary approach and the impact on clinical outcomes.

**Methods:**

This retrospective, single-center study was based on medical records and (FDG-PET)-CT images among gastric/GEJ cancer patients undergoing perioperative FLOT chemotherapy.

**Results:**

Between 2016 and 2021, 46 gastric/GEJ cancer patients started perioperative FLOT at our tertiary cancer center (Salzburg, Austria). At a median follow-up of 32 months median PFS was 47.4 months and median OS was not reached. The skeletal muscle index (SMI, cm^2^/m^2^) turned out to be the only body composition parameter with a statistically significant decrease during pre-operative FLOT (51.3 *versus* 48.8 cm^2^/m^2^, *p* = 0.02). Neither pre-FLOT body mass index (BMI), nor SMI had an impact on the duration of pre-operative FLOT, the time interval from pre-operative FLOT initiation to surgery, the necessity of pre-operative or post-operative FLOT de-escalation or the likelihood of the start of postoperative chemotherapy. Pre-FLOT BMI (overweight *versus* normal, HR: 0.11, 95% CI: 0.02–0.65, *p* = 0.02) and pre-FLOT SMI (sarcopenia *versus* no sarcopenia, HR: 5.08, 95% CI: 1.27–20.31, *p* = 0.02) were statistically significantly associated with PFS in the multivariable analysis.

**Conclusion:**

The statistically significant SMI loss during pre-operative FLOT and the meaningful impact of baseline SMI and BMI on PFS argue for the implementation of a nutritional screening and support program prior to the initiation of pre-operative FLOT in clinical routine.

**Supplementary Information:**

The online version contains supplementary material available at 10.1007/s00432-022-04096-w.

## Introduction

 The phase 3 AIO FLOT4 trial comparing perioperative chemotherapy with FLOT (fluorouracil, oxaliplatin, docetaxel, leucovorin) *versus* ECF/ECX (epirubicin, cisplatin, fluorouracil or capecitabine) demonstrated a statistically significant improvement of median overall survival (OS) from 35 to 50 months (hazard ratio (HR): 0.77) and since then has become the standard regimen for perioperative chemotherapy in locally advanced, operable gastric or gastro-esophageal junction (GEJ) cancer. (National Comprehensive Cancer Network; Smyth et al 2016; Al-Batran et al 2019; Moehler et al 2019) The feasibility of perioperative FLOT in clinical practice outside a clinical trial has been confirmed in the Italian multicenter observational prospective RealFLOT study. (Giommoni et al 2021) Post-gastrectomy weight loss of 8–13% (Heneghan et al 2015; Davis et al 2016) is peaking 6 to 12 months after surgery (Davis et al 2016) and negatively impacts health-related quality of life (QoL). (Climent et al 2017) In this regard, early individualized nutritional support for gastrectomy candidates undergoing perioperative chemotherapy may counteract weight loss. (Rosania et al 2016) Apart from impacting QoL, early weight loss during palliative systemic therapy has already been proven as a negative prognostic factor in advanced gastric/GEJ cancer in the palliative setting. (Mansoor et al 2021) The association between pre-operative underweight (Komatsu et al 2018) as well as post-gastrectomy weight loss (Kubo et al 2016; Lee et al 2016) and worse clinical outcome has also been corroborated in locoregional disease before the establishment of perioperative FLOT as the standard therapeutic approach.

Besides anthropometric parameters such as body weight or the body mass index (BMI), CT-based body composition parameters such as e.g. the skeletal muscle index (SMI) can be easily calculated from routinely acquired CT images (Gomez-Perez et al 2016; Huemer et al 2019) and harbor the potential to serve as prognostic tools among gastric cancer patients undergoing gastrectomy. (Kim et al 2020) In addition, previous studies suggested FDG-PET/CT as an effective non-invasive tool for the prediction of the outcome of the disease. (Vallbohmer et al 2010; Goodman et al 2021) It has been reported that 18F-FDG-uptake in the neck and supraclavicular regions represents activated brown adipose tissue (BAT), which could have a close correlation with clinicopathological features of cancer patients. (Fujii et al 2017).

Data on the short- and long-term impact of the FLOT protocol (Al-Batran et al 2019) on body composition dynamics and in turn the impact of body composition dynamics on clinical outcome are sparse. (Rinninella et al 2021) The aim of this retrospective single-center analysis was to evaluate baseline anthropometric, CT-based and FDG-PET-based body composition parameters as well as short- and long-term dynamics and their impact on perioperative management and on clinical outcome in gastric/GEJ cancer patients undergoing perioperative FLOT therapy with curative intent.

## Patients and methods

### Patients

This retrospective analysis was approved by the Ethics Committee of the provincial government of Salzburg, Austria, on 04 September 2018 (protocol number: 415-EP/73/789–2018). Patients with histologically confirmed gastric/GEJ adenocarcinoma with a clinical stage cT2 or higher and/or nodal positive stage (cN +) according to the 7th or 8th Edition of the International Union against Cancer tumour-node-metastasis classification undergoing perioperative chemotherapy with the FLOT protocol (Al-Batran et al 2019) in curative intent were included.

### Objectives

The primary study objective was to evaluate baseline anthropometric, CT-based and FDG-PET-based body composition parameters as well as short-term and long-term dynamics. Secondary objectives were to evaluate the impact of baseline BMI and SMI as well as their dynamics on clinical outcome (progression-free survival (PFS), OS). Furthermore, the impact of baseline BMI and SMI on the feasibility of this multidisciplinary therapeutic approach was investigated:

- 1. Duration of pre-operative FLOT.
- 2. Time interval from FLOT start to surgery.
- 3. Number of pre-operative chemotherapy cycles.
- 4. Necessity of dose-reductions during pre-operative FLOT.
- 5. Feasibility of postoperative FLOT continuation.
- 6. Number of post-operative chemotherapy cycles.
- 7. Necessity of dose-reductions during post-operative chemotherapy.

### Anthropometric body composition parameters

Body weight (kg) and BMI (kg/m^2^) were assessed at four different time points: 1) at the start of pre-operative FLOT; 2) at the end of pre-operative FLOT; 3) at the start of post-operative FLOT; 4) one year after surgery (time window: 10 to 14 months). The BMI-based nutritional status was divided into the following World Health Organization categories: underweight (< 18.5 kg/m^2^), normal (18.5–24.9 kg/m^2^), overweight (≥ 25–29.9 kg/m^2^), and obese (≥ 30 kg/m^2^).

### CT-based body composition parameters

Two radiologists—one board certified with seven years of experience in oncologic imaging (radiologist 1), one in the third year of training (radiologist 2)—independently assessed skeletal muscle parameters of all included patients by measuring skeletal muscle area (SMA, cm^2^) (Gomez-Perez et al 2016), transverse psoas muscle thickness (TPMT, mm) (Gu et al 2018), psoas muscle area (PMA, mm^2^) (Peng et al 2012) and psoas muscle perimeter (PMP, mm) at three different time points: 1) initial diagnosis (prior to pre-operative FLOT), 2) prior to surgery (after pre-operative FLOT), 3) one year after surgery (time window: 10 to 14 months). The radiologists were blinded to all clinical, histological and laboratory data. Measurements were obtained on axial CT scans of the abdomen performed on a multidetector CT scanner with a patient size‐adapted tube voltage (80‐120 kVp) and active tube current modulation. All imaging data were acquired on either unenhanced or portal venous phase enhanced axial CT images using a soft tissue kernel with a slice thickness of 3 mm and a reconstruction interval of 2 mm. SMA, TPMT, PMA and PMP were calculated in all patients at the level of the third lumbar vertebral body, where both transverse processes were depictable. Measurement of SMA was performed using a free ImageJ software provided by the National Institutes of Health (https://imagej.nih.gov/ij/; Version 1.51): The abdominal perimeter for waist circumference was measured within an attenuation range of –250 to 1000 Hounsfield units (HU), the outer and inner musculature perimeter and the lumbar vertebra within an attenuation range of –29 to 150 HU. The SMA was calculated by subtracting the inner musculature perimeter and the lumbar vertebra from the outer musculature perimeter (Online Resource 1). Adjustment of the SMA for the square of the height yielded the SMI (cm^2^/m^2^). Sarcopenia was defined by established sex-specific cut-offs for SMI which are for men: < 52.4 cm^2^/m^2^ and for women: < 38.5 cm^2^/m^2^. (Prado et al 2008) Sarcopenic obesity was defined as sarcopenia in combination with a BMI ≥ 25 kg/m^2^.

TPMT, PMA and PMP were measured on a picture archiving and communication system (PACS, workstation, Impax; Agfa, Mortsel, Belgium). TPMT was defined as the greatest transverse diameter of the right psoas muscle perpendicular to the long axis (anterior–posterior oblique) of the psoas muscle (Online Resource 1). (Gu et al 2018) PMP was measured by outlining the circumference of the right psoas muscle, which also yielded PMA (Online Resource 1). (Peng et al 2012) Results were adjusted for the square of the height and are shown as mm/m^2^ for TPMT and PMP, and as mm^2^/m^2^ for PMA.

### FDG-PET-based body composition parameters

Distribution of BAT differs between patients concerning age, gender and underlying diseases. BAT may be visualized in certain areas including lower neck, supraclavicular, paravertebral, perirenal, axillary, retroperitoneal, perivascular and mediastinal regions. Regarding the fact that reactive BAT is more visible in lower cervical, supraclavicular and upper axillary regions on FDG-PET/CT images, these specific areas were selected for this analysis. Volume of interest (VOI) was drawn manually in the right cervical, supraclavicular and axillary region from the base of the skull to the region of the second rib and quantitative parameters including standardized uptake value (SUV) max, SUVmean, volume, and mean HU were reported. Areas affected by artifacts like beam hardening due to metal devices were excluded. Areas of cervical, axillary or supraclavicular lymph nodes were also manually excluded. Volume measurements were limited to areas adjacent to the muscles with high FDG uptake (Online Resource 1).

### Tumor regression grade

The biopsy-based gastric/GEJ tumor diagnosis prior to chemotherapy, the surgical resectate-based neoadjuvant TNM staging as well as the tumor regression grade according to the Becker criteria (Becker et al 2003) after pre-operative FLOT was assessed by two consultant pathologists (EK and DN). The Becker classification of histopathologic regression was based on the estimation of the percentage of vital tumor tissue in relation to the macroscopically identifiable tumor bed (ranging from 1a–3). Grade 1a defined a pathologic complete remission whereas more than 50% remaining residual tumor corresponded to grade 3. (Becker et al 2003).

### Statistical analysis

Baseline characteristics were compared using crosstabulation together with the chi-squared test in the case of categorical data. Continuous data were summarized using medians and ranges and compared between groups with the Mann–Whitney test. Correlations were tested using the Spearman test. Uni- and multivariable analyses were based on COX proportional hazard models. For multivariable analysis covariable selection, a backward stepwise procedure was performed using the Akaike information criterion (AIC) as a selection criterion. (Heinze et al 2018) Kaplan–Meier survival curves together with log-rank testing were used to evaluate PFS and OS. PFS was calculated from the date of treatment start until radiologically confirmed progression or death. OS was calculated from the date of treatment start until death from any cause. Patients alive at the last contact were censored. IBM SPSS Statistics version 27 (Armonk, NY, US) and the statistical software environment R (version 4.1.2, survival and MASS package) were used for statistical analyses.

## Results

### Baseline characteristics

Between May 2016 and March 2021, 46 gastric/GEJ cancer patients started perioperative chemotherapy with the FLOT protocol in curative intent. Baseline characteristics are depicted in Online Resource 2.

After a median follow-up of 32 months, the median PFS was 47.4 months (95% CI: 38.8-NA, Online Resource 3a) while the median OS was not reached (95% CI: 48.6-NA, Online Resource 3b). The median duration from the first to the last pre-operative FLOT cycle was 44 days (range: 14–86) and the median time interval from pre-operative FLOT initiation to curative surgery was 85 days (range: 49–120). One patient received seven pre-operative FLOT cycles due to the diagnosis of an acute pulmonary embolism as bridging therapy until eligibility for curative surgery. Forty-three patients (93%) received all four allocated pre-operative FLOT cycles. Three patients discontinued pre-operative chemotherapy due to inadequately controlled chemotherapy side effects despite proper supportive care. Five patients (11%) achieved a pathologic complete remission (pCR) and 38 patients (83%) continued post-operative FLOT therapy (Online Resource 2 & 4).

### Anthropometric body composition parameters and dynamics

At the start of pre-operative FLOT no patients were classified as “underweight” whereas 27 patients (59%) were classified as “overweight” or “obese” according to the BMI-based WHO classification. However, only seven patients (16%) fulfilled the definition of “sarcopenic obesity”. No significant changes in median body weight (80 *versus* 80 kg, *p* = 0.48) or median BMI (26.2 *versus* 26.0 kg/m^2^, *p* = 0.51) were observed between the start and the end of pre-operative FLOT. However, a statistically significant and clinically meaningful short-term decline in median body weight (80 *versus* 72 kg, *p* < 0.001) and median BMI (26.0 *versus* 23.6 kg/m^2^, *p* < 0.001) were found between the last pre-operative FLOT cycle and the start of post-operative FLOT after the surgical procedure, which was the main cause of short-term and long-term body weight and BMI dynamics (Table 1).

### CT- and FDG-PET-based body composition parameters and dynamics

A strong and statistically highly significant interobserver correlation was found for each CT-based body composition parameter (Online Resource 5).

At the time point of gastric/GEJ cancer diagnosis, 19 patients (43%) fulfilled the criterion of sarcopenia. The latter percentage rose to 62% one year after surgery. Among CT- and FDG-PET-based body composition parameters, only the SMI turned out to significantly decrease between baseline imaging studies prior to pre-operative FLOT and imaging studies after pre-operative FLOT prior to surgery (51.3 *versus* 48.8 cm^2^/m^2^, *p* = 0.02). Besides SMI, only TPMT and PMA significantly decreased between baseline imaging studies and imaging studies one-year post-surgery and between pre-operative imaging studies and one-year post-surgery (time window: 10–14 months, respectively) (Table 2).

### Correlation between baseline anthropometric, CT- and FDG-PET-based body composition parameters

The anthropometric baseline body composition parameters (body weight and BMI) showed a statistically significantly weak to moderate positive correlation with CT-based body composition parameters. Among FDG-PET-based body composition parameters, body weight (*r* = 0.683, *p* < 0.001) and BMI (0.708, *p* < 0.001) only showed a moderate positive correlation with BAT volume and a weak positive correlation with SUVmax (*r* = 0.373, *p* = 0.05 and *r* = 0.424, *p* = 0.03, respectively) whereas the moderate correlation with BAT HU was inverse (*r* = –0.632, *p* < 0.001 and *r* = –0.540, *p* = 0.003, respectively). A statistically significant correlation between SMI and anthropometric as well as each CT-based body composition parameter was seen whereas there was no correlation with FDG-PET-based body composition parameters (Online Resource 6).

### Impact of baseline body mass index and skeletal muscle index on the feasibility of perioperative FLOT

Neither baseline BMI (overweight *versus* non-overweight), nor baseline SMI (sarcopenia *versus* no sarcopenia) had an impact on the duration of pre-operative FLOT therapy or pre-operative FLOT initiation to surgery, probability of postoperative chemotherapy continuation, the necessity of pre- or postoperative dose reductions or the number of pre- or post-operative chemotherapy cycles (Online Resource 7).

### Impact of body composition parameters on clinical outcome

#### Univariable analysis

**Fig. 1:**
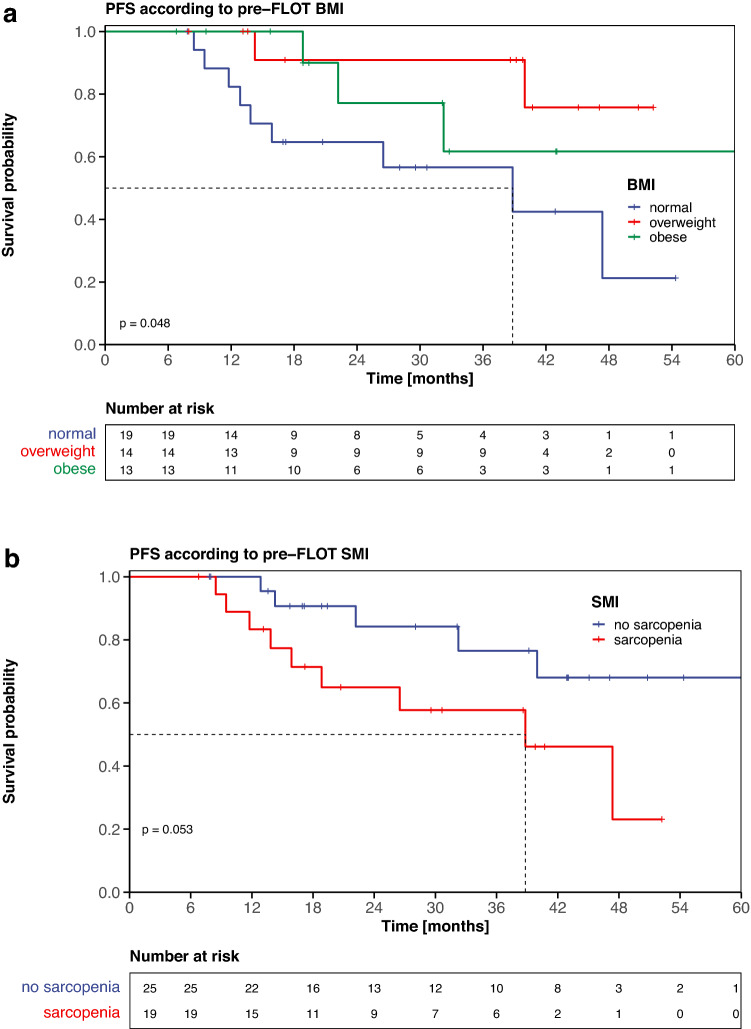
Progression-free survival (PFS) according to anthropometric and CT-based body composition parameters**. a** Kaplan-Meier curves (PFS) according to pre-FLOT BMI. **b** Kaplan-Meier curves (PFS) according to pre-FLOT SMI. The tick marks on the curve represent censored patients

The influence of baseline characteristics, tumor characteristics, baseline body composition parameters and their dynamics on PFS and OS in the univariable analysis is depicted in Table 3:

Higher pre-FLOT body weight (HR: 0.96, *p* = 0.04) and pre-FLOT overweight (*versus* normal, HR: 0.20, *p* = 0.04, Fig. 1a) were associated with superior PFS in the univariable analysis. Higher pre-FLOT SMI (HR: 0.95, *p* = 0.09) and tumors in the GEJ (*versus* stomach, HR: 0.34, *p* = 0.07) showed a trend towards superior PFS whereas pre-FLOT sarcopenia (*versus* no sarcopenia, HR: 2.83, *p* = 0.06; Fig. 1b) showed a trend towards inferior PFS. BMI dynamics (increase *versus* decrease, HR: 0.63, *p* = 0.38) and SMI dynamics (increase *versus* decrease, HR: 1.56, *p* = 0.41) during pre-operative FLOT did not influence PFS. Neither the abovementioned baseline body composition parameters nor their dynamics had an impact on OS in univariable analysis.

#### Multivariable analysis

##### Progression-free survival

**Fig. 2:**
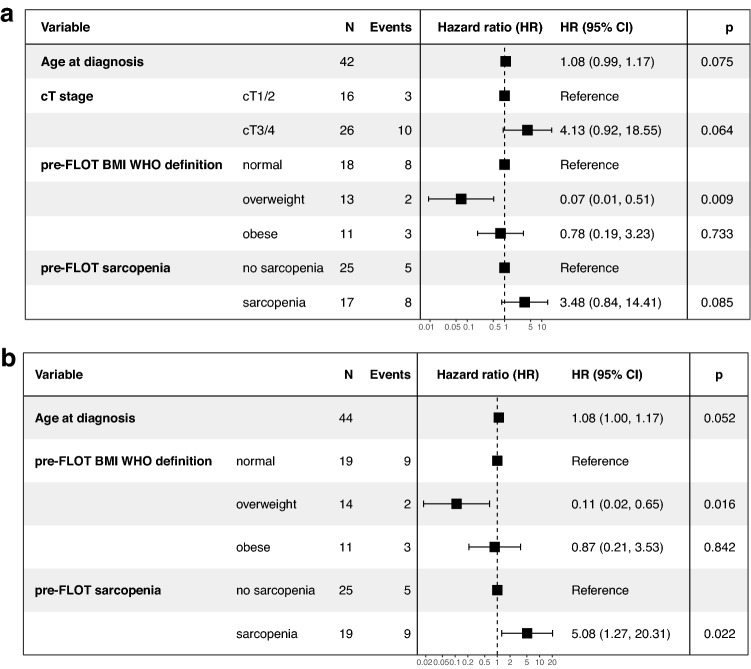
Progression-free survival multivariable analysis – Forest Plots. BMI: body mass index, HR: hazard ratio, 95% CI: 95% confidence interval

Based on a backward stepwise selection procedure and due to the limited number of events (disease recurrence/death: *n* = 14) two PFS multivariable analysis models were investigated:

Model 1 (stepwise selection procedure included all covariables):

The following covariables were selected for PFS multivariable analysis: age at diagnosis, cT stage (cT 3/4 *versus* cT 1/2), pre-FLOT BMI (normal *versus* overweight *versus* obese) and pre-FLOT SMI (sarcopenia *versus* no sarcopenia). Pre-FLOT BMI (overweight *versus* normal weight, HR: 0.07, 95% CI: 0.01–0.51, *p* = 0.01) was statistically significantly associated with PFS (Fig. 2a).

Model 2 (stepwise selection procedure included only at baseline available covariables except for cT and cN stage).

The following covariables were selected for PFS multivariable analysis:

age at diagnosis, pre-FLOT BMI (normal *versus* overweight *versus* obese) and pre-FLOT SMI (sarcopenia *versus* no sarcopenia). Pre-FLOT BMI (overweight *versus* normal weight, HR: 0.11, 95% CI: 0.02–0.65, *p* = 0.02) and pre-FLOT SMI (sarcopenia *versus* no sarcopenia, HR: 5.08, 95% CI: 1.27–20.31, *p* = 0.02) were statistically significantly associated with PFS (Fig. 2b).

##### Overall survival

Based on a backward stepwise selection procedure, no multivariable COX regression model could be selected including significant covariables.

## Discussion

In this analysis, we report on anthropometric and imaging-based body composition parameters and dynamics in a homogeneous real-world gastric/GEJ cancer patient cohort undergoing perioperative FLOT chemotherapy. We found a clinically relevant SMI loss (Table 2) between baseline imaging studies prior to pre-operative FLOT and after pre-operative FLOT prior to surgery. In contrast, anthropometric body composition parameters (Table 1) were not affected during pre-operative chemotherapy. Neither baseline BMI nor SMI had an impact on the feasibility of perioperative FLOT or the timing of curative surgery (Online Resource 7). In this regard, it is noteworthy that Rinninella et al. reported on the body composition dynamics (e.g. BMI and SMI) and the impact on the feasibility and toxicity among gastric cancer patients undergoing perioperative FLOT in a small Italian cohort study (n = 26). No association between body composition dynamics and chemotherapy delay, toxicity or the probability of postoperative chemotherapy completion was seen. (Rinninella et al 2021).

**Table 1:** Anthropometric body composition parameters and dynamics

	Pre-operative FLOT start (%)	Pre-operative FLOT end (%)	Post-operative FLOT start (%)	1 year post-surgery^a^ (%)
**BMI WHO definition**				
Underweight (<18.5 kg/m^2^)	0 (0)	2 (4)	2 (5)	5 (13)
Normal (18.5-24.9 kg/m^2^)	19 (41)	17 (38)	22 (58)	23 (61)
Overweight (≥25-29.9 kg/m^2^)	14 (31)	14 (31)	9 (24)	8 (21)
Obese (≥30 kg/m^2^)	13 (28)	12 (27)	5 (13)	2 (5)
**Sarcopenic obesity** (Sarcopenia + BMI ≥25 kg/m^2^)				
yes	7 (16)	9 (20)	–	2 (6)
no	37 (84)	36 (80)		34 (94)
NA	2	1		10
**Median weight **(kg)	80	80	72	70
(range: minimum-maximum)	46–128	41–122	47–118	38–108
	*P* = 0.48 80 pre-operative FLOT start vs 80 pre-operative FLOT end			
	*P* = 0.003* 72 post-operative FLOT start vs 70 1 year post-surgery			
	*P *< 0.001* 80 pre-operative FLOT end vs 72 post-operative FLOT start			
	*P *< 0.001* 80 pre-operative FLOT end vs 70 1 year post-surgery			
	P < 0.001* 80 pre-operative FLOT start vs 70 1 year post-surgery			
**Median BMI **(kg/m^2^**)**	26.2	26	23.6	22.1
(range: minimum-maximum)	19-41	17.1–38.4	15.7–35.5	15.2–34.9
	*P *= 0.51 26.2 pre-operative FLOT start vs 26.0 pre-operative FLOT end			
	*P *= 0.002* 23.6 post-operative FLOT start vs 22.1 1 year post-surgery			
	*P *< 0.001* 26.0 pre-operative FLOT end vs 23.6 post-operative FLOT start			
	*P *< 0.001* 26.0 pre-operative FLOT end vs 22.1 1 year post-surgery			
	*P *< 0.001* 26.2 pre-operative FLOT start vs 22.1 1 year post-surgery			

^a^Time window: 10–14 months

^*^Statistically significant (*p* < 0.05)

BMI: body mass index

Our single-center real-world cohort of gastric/GEJ cancer patients undergoing perioperative FLOT with curative intent is representative concerning clinical outcome (Online Resource 3 & 4) and pre-operative FLOT compliance when compared to the FLOT4 and RealFLOT study (Online Resource 4). In our retrospective study, the majority of patients (83%) started postoperative chemotherapy with FLOT in this cross-trial comparison, however, this finding was not influenced by baseline BMI or SMI (Online Resource 7). Previous reports in the pre-FLOT era described a higher frequency of dose-limiting toxicities (Tan et al 2015) and a higher likelihood of neoadjuvant chemotherapy termination among gastric/GEJ cancer patients with baseline sarcopenia. (Palmela et al 2017) It is noteworthy, that the SMI-based definition of sarcopenia by Martin’s definition in the latter study (women: SMI < 41 cm^2^/m^2^, men with a BMI < 25 kg/m^2^: < 43 cm^2^/m^2^, men with a BMI ≥ 25 kg/m^2^: < 53 cm^2^/m^2^) (Palmela et al 2017) differed from the sarcopenia definition in our analysis (women: < 38.5 cm^2^/m^2^, men: < 52.4 cm^2^/m^2^). (Prado et al 2008) We decided to choose Prado’s sarcopenia definition in our analysis as Martin’s sarcopenia definition uses discontinuous cut-offs for men although the SMI is already normalized by the square of the height. (Taguchi et al 2020) In contrast to the aforementioned studies, neither baseline BMI, nor SMI had an influence on the number of pre- or postoperative chemotherapy cycles or on the necessity of dose-reductions in our cohort (Online Resource 7). Although no patient in our cohort fulfilled the BMI-based criterion of “underweight” at baseline, 43% were already identified as sarcopenic. Awad et al. reported a slightly higher incidence of baseline sarcopenia (57%) prior to the initiation of neoadjuvant chemotherapy in their retrospective analysis of esophagogastric cancer patients. (Awad et al 2012) The discrepancy between BMI-defined underweight and SMI-defined sarcopenia and only a weak to moderate positive correlation between anthropometric and CT-based body composition parameters (Online Resource 6) in our analysis corroborate our recommendation to integrate SMI assessment into a standardized nutritional screening and support program among gastric/GEJ cancer patients undergoing perioperative chemotherapy. CT-based body composition parameters (e.g. SMI) can be easily calculated from routinely acquired CT images and the statistically highly significant and strong interobserver correlation in our analysis (Online Resource 5) shows the feasibility and reproducibility of this approach in clinical practice.

Based on the negative correlation between BAT and BMI in previous reports, BAT has been proposed as a surrogate parameter for the nutritional status in healthy volunteers (van Marken Lichtenbelt et al 2009) as well as in cancer patients. (Rousseau et al 2006) Obese adipose tissue is linked to an autoinflammatory condition and as a consequence modulates the tumor microenvironment. (Santander et al 2015) Furthermore, BAT expansion drives unrestrained lipolysis and increased energy expenditure thereby contributing to cancer cachexia. (Huang et al 2011; Vaitkus and Celi 2017) The availability of sequential pre-FLOT and pre-operative FDG-PET/CT images in 20 patients enabled us to investigate BAT body composition dynamics during pre-operative FLOT (Online Resource 1). However, we did not detect any clinically relevant dynamics of FDG-PET-based BAT body composition parameters during pre-operative FLOT (Table 2). This might be due to the limitations caused by manual quantitative analysis in selected regions and the fact that multiple factors may affect BAT activity on FDG-PET/CT. Due to the latter findings and the circumstance that FDG-PET/CT imaging is not routinely performed for initial staging in clinical practice (rather in selected cases) (National Comprehensive Cancer Network; Smyth et al 2016), the integration of FDG-PET-based BAT body composition parameters into nutritional screening and support programs seems neither of benefit nor feasible in clinical routine.

**Table 2:** CT and FDG-PET-based body composition parameters and dynamics

	Pre-FLOT CT/PET-CT (%)	Pre-operative CT/PET-CT (%)	1 year post-surgery^a^(%)
**Median skeletal muscle index** (cm^2^/m^2^**)** (range: minimum-maximum)	51.3	48.8	46.6
	34.1–72.7	31.7–73.2	33.2–62.2
	*n*=44	*n*=45	*n*=34
	*P*=0.02* 51.3 pre-FLOT vs 48.8 pre-operative		
	*P*=0.01* 48.8 pre-operative vs 46.6 1 year post-surgery		
	*P*<0.001* 51.3 pre-FLOT vs 46.6 1 year post-surgery		
**Sarcopenia** male: SMI <52.4 cm^2^/m^2^ female: SMI <38.5 cm^2^/m^2^	43.20%	51.10%	61.80%
	*n*=44	*n*=45	*n*=34
	*P*=0.45^b^ 43.2% pre-FLOT vs 51.1% pre-operative		
	*P*=0.35^b^ 51.1% pre-operative vs 61.8% 1 year post-surgery		
	*P*=0.10^b^ 43.2% pre-FLOT vs 61.8% 1 year post-surgery		
**Median transverse psoas muscle thickness** (mm/m^2^) (range: minimum-maximum)	9.3	9.2	8.2
	5.6–15.6	5.5–15.2	4.5–11.4
	*n*=44	*n*=45	*n*=34
	*P*=0.54 9.3 pre-FLOT vs 9.2 pre-operative		
	*P*=0.008* 9.2 pre-operative vs 8.2 1 year post-surgery		
	*P*=0.006* 9.3 pre-FLOT vs 8.2 1 year post-surgery		
**Median psoas muscle area** (mm^2^/m^2^) (range: minimum-maximum)	311.7	299.7	269.4
	116.1–596.9	90.7–567.9	117.8–421.3
	*n*=44	*n*=45	*n*=34
	*P*=0.13 311.7 pre-FLOT vs 299.7 pre-operative		
	*P*=0.01* 299.7 pre-operative vs 269.4 1 year post-surgery		
	*P*=0.004* 311.7 pre-FLOT vs 269.4 1 year post-surgery		
**Median psoas muscle perimeter** (mm/m^2^) (range: minimum-maximum)	42.1	42.2	42.8
	29.0–55.0	34.3–52.5	33.1–48.5
	*n*=44	*n*=45	*n*=34
	*P*=0.71 42.1 pre-FLOT vs 42.2 pre-operative		
	*P*=0.24 42.2 pre-operative vs 42.8 1 year post-surgery		
	*P*=0.89 42.1 pre-FLOT vs 42.8 1 year post-surgery		
**BAT volume** (range: minimum-maximum)	102	115	NA
	36.0–243.0	21.6–238.0	
	*n*=28	*n*=24	
	*P*=0.59 102.0 pre-FLOT vs 115 pre-operative		
**BAT SUV max** (range: minimum-maximum)	1.6 1.10–2.57 *n*=28	1.57 0.93–2.91 *n*=24	NA
	*P*=0.31 1.60 pre-FLOT vs 1.57 pre-operative		
**BAT SUV mean** (range: minimum-maximum)	0.63	0.63	NA
	0.50–61.00	0.35–64.00	
	*n*=28	*n*=24	
	*P*=0.56 0.63 pre-FLOT vs 0.63 pre-operative		
**BAT HU** (range: minimum-maximum)	–95.9	–91.3	NA
	–123.0–(–)67.0	–132.0–(–)72.5	
	*n*=28	*n*=24	
	*P*=0.08 − 95.9 pre-FLOT vs − 91.3 pre-operative		

^b^Chi-square test

^*^Statistically significant (*p* < 0.05)

*BAT* brown adipose tissue, *NA* not available, *SMI* skeletal muscle index

The impact of baseline body composition parameters and/or their dynamics on clinical outcome has been investigated by a large number of authors in heterogeneous patient cohorts in regard to the application of chemotherapy (none, neoadjuvant, adjuvant, perioperative, not reported) (Kubo et al 2016; Mirkin et al 2017; Palmela et al 2017; Komatsu et al 2018; Park et al 2018; Kim et al 2020) and in regard to the chemotherapy protocol (Mirkin et al 2017; Palmela et al 2017; Park et al 2018; Kim et al 2020) prior to the establishment of FLOT as a new standard, therefore, the interpretation of the conflicting results is challenging. Mirkin et al. investigated the influence of sarcopenia on clinical outcome among gastric cancer patients (*n* = 36) undergoing neoadjuvant chemotherapy with various protocols. Despite the same definition of SMI-based sarcopenia, fewer patients (19%) presented with initial sarcopenia when compared to our findings (43%) and the authors did not find an impact of sarcopenia on clinical outcome. (Mirkin et al 2017) We assume that the low patient number and the low incidence of baseline sarcopenia in the latter study considerably influenced the reported findings concerning the clinical outcome.

Baseline BMI (overweight *versus* normal; HR: 0.07 (Model 1, Fig. 2a), HR: 0.11 (Model 2, Fig. 2b)) and baseline SMI (sarcopenia *versus* no sarcopenia; HR: 5.08 (Model 2, Fig. 2b)) had an independent and clinically meaningful impact on PFS in our cohort. Due to the considerable discordance between clinical and pathologic staging of resectable, locally advanced gastric cancer (Papageorge et al 2021), cT stage and cN stage were excluded from the backward stepwise covariable selection procedure for PFS multivariable analysis in Model 2 (Fig. 2b).

Although neither short-term BMI nor SMI dynamics had an impact on clinical outcome (Table 3), the SMI decline during pre-operative FLOT indicates an early onset of muscle loss already in the pre-operative time period (Table 2). Furthermore, our findings of a considerable long-term decrease in anthropometric (Table 1) and CT-based (Table 2) body composition parameters – mainly caused by the surgical procedure—argue for the establishment of standardized early nutritional screening and support programs for gastric/GEJ cancer patients undergoing perioperative chemotherapy with the FLOT protocol nowadays. The effect of oral nutritional support on the nutritional status (e.g. reduction of body weight loss) in patients with gastric cancer has been corroborated in a meta-analysis by Rinninella et al. (Rinninella et al 2020) However, it is noteworthy, that the oral nutritional intervention in the vast majority of included randomized, controlled trials were in close temporal proximity to the surgery (Rinninella et al 2020). Data from completed and ongoing trials investigating the effect of nutritional interventions during perioperative chemotherapy in gastric/GEJ cancer patients are limited. (Mulazzani et al 2021).

**Table 3:** Progression-free survival and overall survival univariable analysis

**Progression-free survival**				
Parameter		HR	95% CI	p-value
Age at diagnosis	years	1.01	0.96–1.07	0.74
Pre-FLOT body weight	kg	0.96	0.93–1.00	0.04*
Pre-FLOT BMI	kg/m^2^	0.90	0.80–1.02	0.11
Pre-FLOT BMI WHO	normal			
	overweight	0.20	0.04–0.92	0.04*
	obese	0.45	0.12–1.66	0.23
Pre-operative BMI dynamics	decrease			
	increase	0.63	0.22–1.79	0.38
Pre-FLOT SMI	cm^2^/m^2^	0.95	0.89–1.01	0.09
Pre-FLOT sarcopenia	no sarcopenia			
	sarcopenia	2.83	0.94–8.50	0.06
Pre-operative SMI dynamics	decrease			
	increase	1.56	0.54–4.55	0.41
Sarcopenic obesity	no			
	yes	0.37	0.05–2.80	0.33
Sex	male			
	female	0.91	0.25–3.29	0.88
Pre-FLOT ECOG PS	0			
	1	0.98	0.34–2.84	0.97
Primary tumor localization	stomach			
	GEJ	0.34	0.10–1.09	0.07
cN stage	negative			
	positive	1.09	0.51–2.31	0.83
cT stage	cT 1/2			
	cT 3/4	2.16	0.86–5.42	0.10
Tumor regression grade (Becker criteria)	1a			
	1b	0.48	0.04–5.40	0.32
	2	3.42	0.41–28.7	0.26
	3	1.91	0.22–16.50	0.56
Postoperative FLOT start	no			
	yes	0.67	0.18–2.47	0.55

**Overall survival**				
Parameter		HR	95% CI	p-value
Age at diagnosis	years	1.01	0.94–1.09	0.76
Pre-FLOT body weight	kg	1.00	0.95–1.04	0.85
Pre-FLOT BMI	kg/m^2^	1.00	0.86–1.15	0.98
Pre-FLOT BMI WHO	normal			
	overweight	0.30	0.03–2.88	0.29
	obese	1.28	0.26–6.41	0.76
Pre-operative BMI dynamics	decrease			
	increase	0.80	0.18–3.60	0.77
Pre-FLOT SMI	cm^2^/m^2^	0.99	0.92–1.08	0.86
Pre-FLOT sarcopenia	no sarcopenia			
	sarcopenia	1.99	0.44–8.96	0.37
Pre-operative SMI dynamics	decrease			
	increase	0.83	0.16–4.38	0.82
Sarcopenic obesity	no			
	yes	0.75	0.09–6.27	0.79
Sex	male			
	female	0.42	0.05–3.63	0.43
Pre-FLOT ECOG PS	0			
	1	1.37	0.29–6.47	0.69
Primary tumor localization	stomach			
	GEJ	0.30	0.06–1.58	0.16
cN stage	negative			
	positive	0.64	0.20–2.05	0.45
cT stage	cT 1/2			
	cT 3/4	3.28	0.73–14.70	0.12
Tumor regression grade (Becker criteria)	1a			
	1b	0.33	0.02–5.42	0.44
	2	2.05	0.21–20.10	0.54
	3	0.90	0.08–10.20	0.93
Postoperative FLOT start	no			
	yes	0.40	0.07–2.21	0.29

^*^statistically significant (*p* < 0.05). *BMI* body mass index, *ECOG* *PS* Eastern Cooperative Oncology Group performance status, *SMI* skeletal muscle index

Bozzetti et al. randomly assigned gastrointestinal cancer patients with a body weight loss of ≥ 10% to ten days of preoperative and nine days of postoperative total parenteral nutrition *versus* a non-interventional control group and the authors reported a reduction in postoperative complications and of postoperative mortality. (Bozzetti et al 2000) The randomized, controlled, multicenter and observer-blinded PERCOG trial investigates whether early additional supplemental parenteral nutrition (starting on the first day of pre-operative chemotherapy) in gastric/GEJ cancer patients can decrease postoperative complications, however, results have not been published yet. (Mueller et al 2017) Kira et al. prospectively compared the impact of parenteral *versus* enteral nutrition (three days before the start of chemotherapy to seven days after chemotherapy completion) on SMI dynamics among esophageal cancer patients undergoing pre-operative chemotherapy with cisplatin, adriamycin and 5-FU. In the latter study, enteral nutrition support proved superior to parenteral nutrition support in terms of a reduction of SMI loss during neoadjuvant chemotherapy (–1.4 *versus* –3.0 cm^2^/m^2^, *p* < 0.001). Patients with a low SMI after neoadjuvant chemotherapy turned out to be more susceptible to postoperative complications. (Kita et al 2021).

Nutritional screening, support and physical activity recommendations during perioperative chemotherapy are hardly covered in the European Society of Medical Oncology guidelines (Smyth et al 2016) or National Comprehensive Cancer Network guidelines (National Comprehensive Cancer Network) whereas current evidence and recommendations concerning nutritional support is discussed in the German S3 gastric/GEJ cancer guidelines in detail. (Moehler et al 2019) A practical guideline covering nutrition in cancer with recommendations for clinical practice is provided by the European Society for Clinical Nutrition and Metabolism. (Muscaritoli et al 2021).

Although the number of patients included in our single center-cohort exceeded the number of patients in the abovementioned analyses (Mirkin et al 2017; Rinninella et al 2021), the sample size (*n* = 46) as well as the number of events (disease recurrence/death: *n* = 14) are a limitation of this study. Furthermore, the follow-up period (median: 32 months) was shorter in comparison to the FLOT4 study (43 months), therefore, the impact of body composition parameters and dynamics on clinical outcome has to be interpreted with caution.

## Conclusions

Sequential CT-based SMI assessments during pre-operative FLOT unmask a clinically relevant skeletal muscle loss whereas dynamics of anthropometric body composition parameters lag behind. Although neither baseline SMI, nor BMI negatively impact the feasibility of perioperative FLOT with curative intent, the latter body composition parameters showed a considerable impact on clinical outcome in our cohort. Our findings corroborate the necessity to consistently implement nutritional screening and support programs prior to the start of perioperative chemotherapy with FLOT in gastric/GEJ cancer patients.

## Availability of data materials

Data are available from the corresponding author upon reasonable request.

## Supplementary Information

Below is the link to the electronic supplementary material.

Supplementary file1 (PDF 420 KB)

Supplementary file2 (PDF 32 KB)

Supplementary file3 (PDF 17 KB)

Supplementary file3 (DOCX 18 KB)
