# Supplementary figures and images for: Body composition dynamics and impact on clinical outcome in gastric and gastro-esophageal junction cancer patients undergoing perioperative chemotherapy with the FLOT protocol
Source: J Cancer Res Clin Oncol. 2022 Jul 21;149(7):3051–64. doi: 10.1007/s00432-022-04096-w (PMC10314876; doi:10.1007/s00432-022-04096-w)

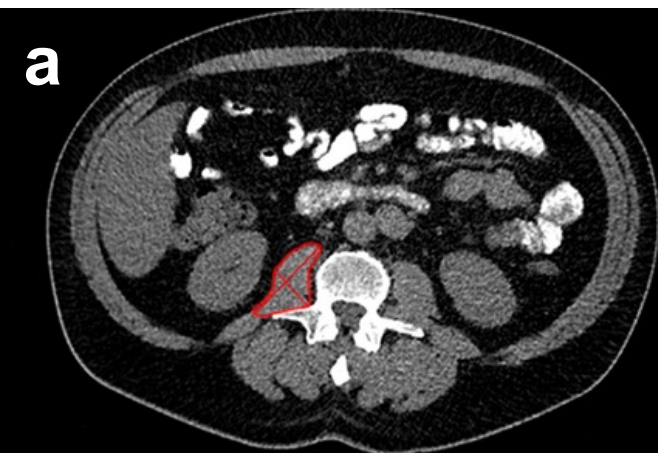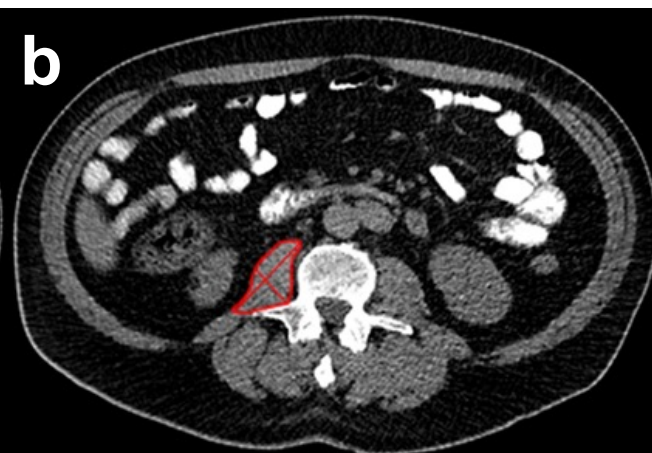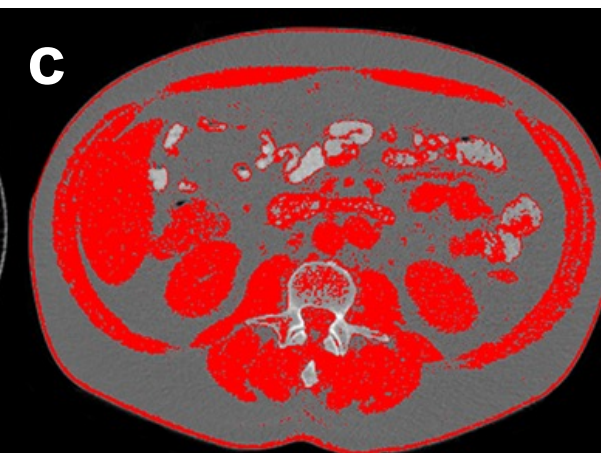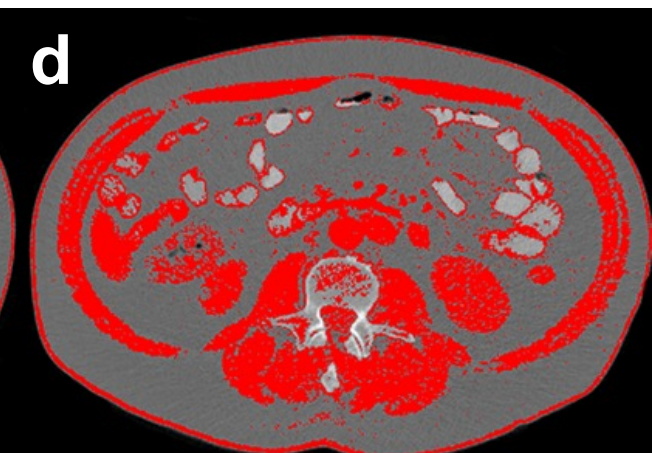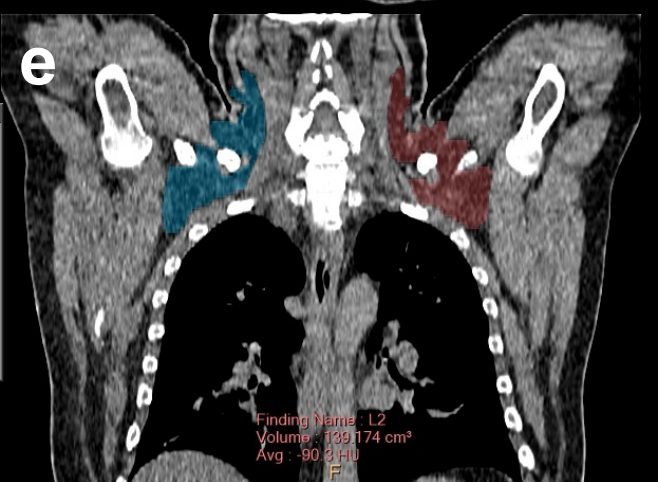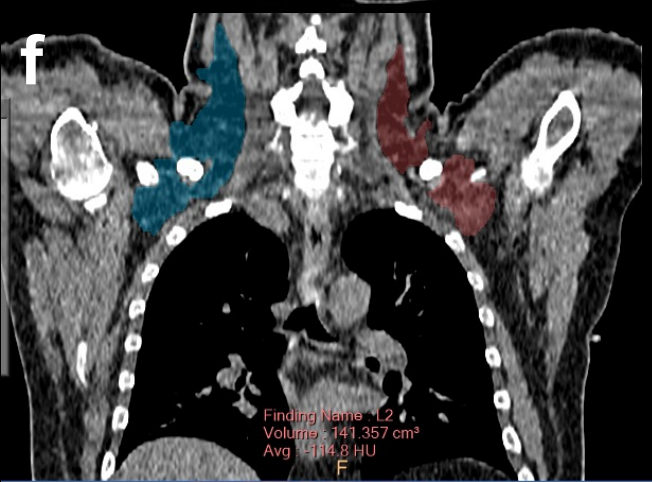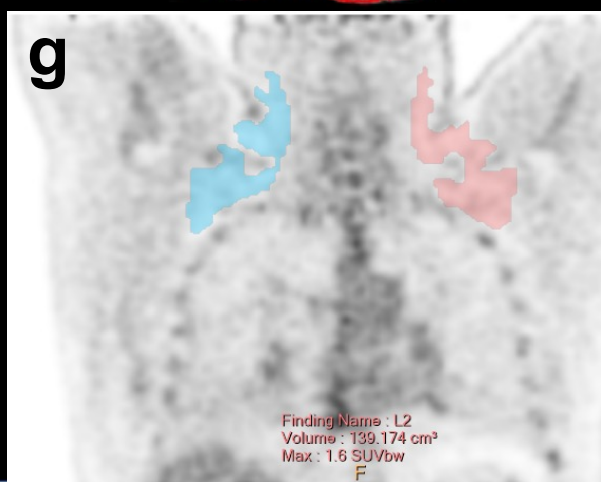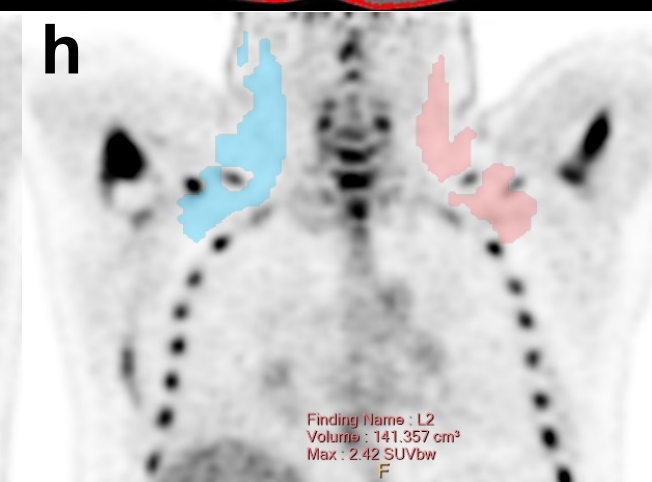

Supplement: Supplementary file 1 — Supplementary file1 (PDF 420 KB) [file 432_2022_4096_MOESM1_ESM.pdf]

**PFS overall cohort**

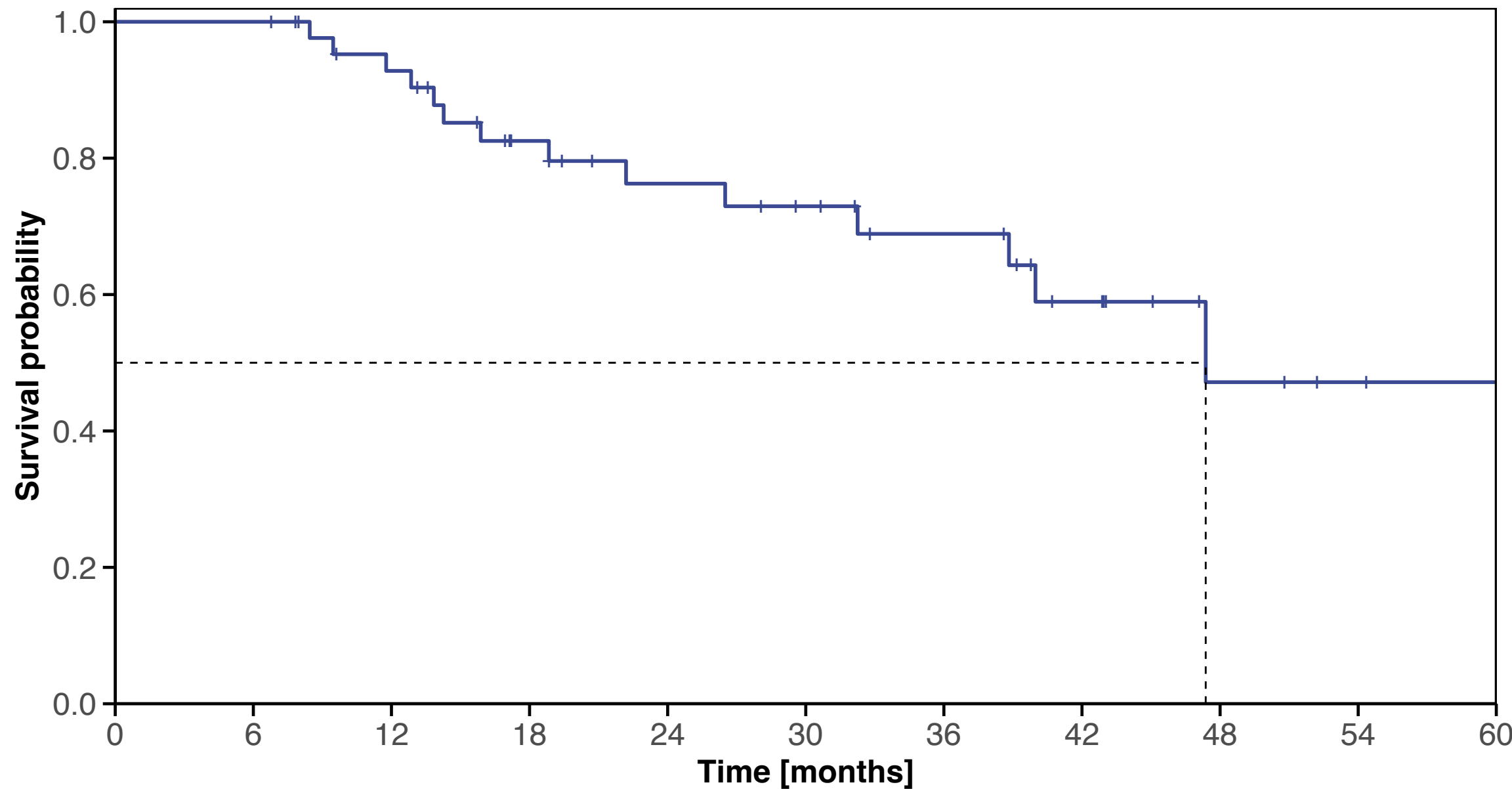

**Number at risk**

|    |    |    |    |    |    |    |    |   |   |   |
|----|----|----|----|----|----|----|----|---|---|---|
| 46 | 46 | 38 | 28 | 23 | 20 | 16 | 10 | 4 | 2 | 1 |
|----|----|----|----|----|----|----|----|---|---|---|

Supplement: Supplementary file 3 — Supplementary file3 (PDF 17 KB) [file 432_2022_4096_MOESM3_ESM.pdf]

**OS overall cohort**

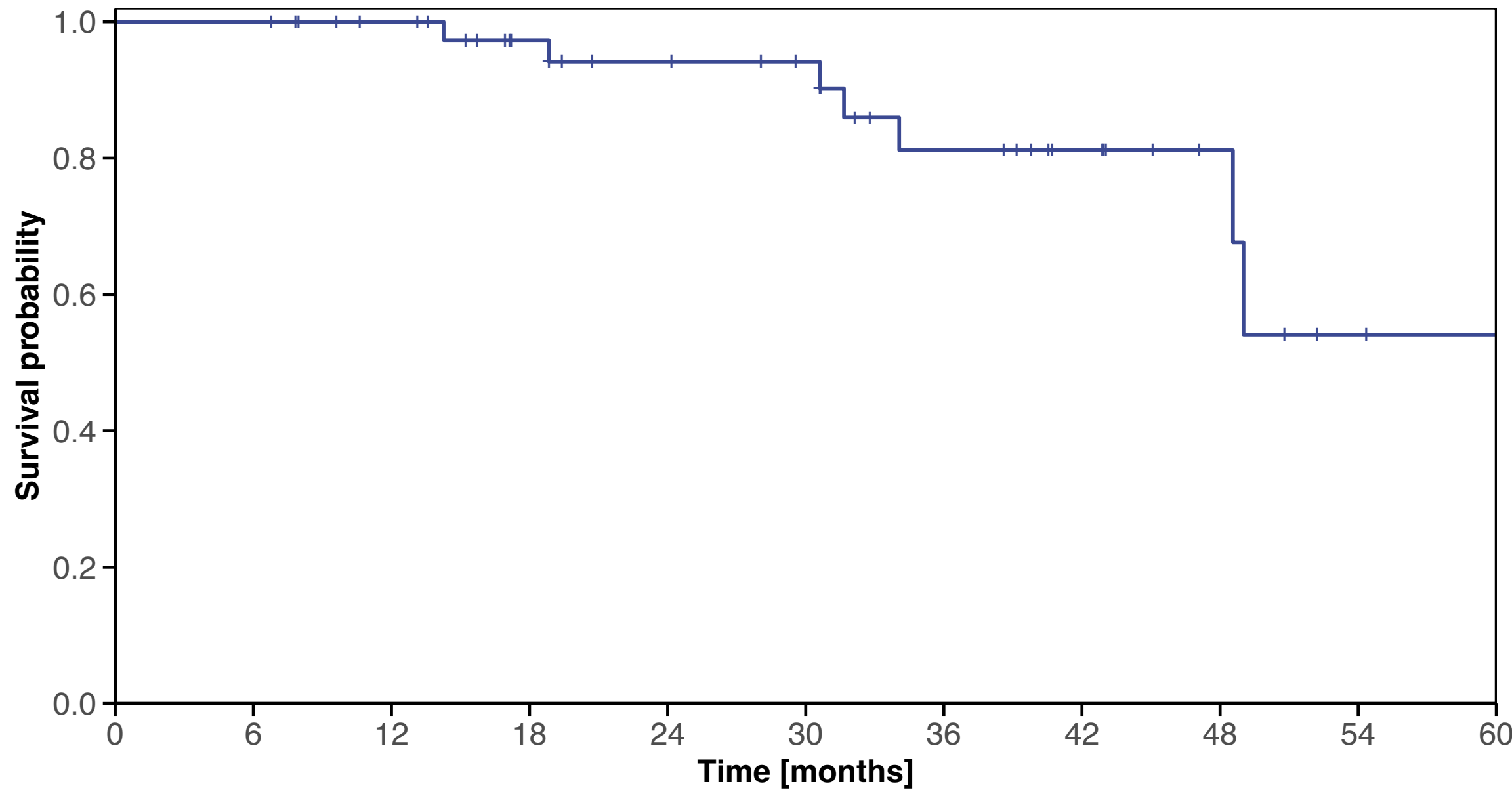

**Number at risk**

|    |    |    |    |    |    |    |    |   |   |   |
|----|----|----|----|----|----|----|----|---|---|---|
| 46 | 46 | 40 | 31 | 27 | 24 | 17 | 12 | 6 | 2 | 1 |
|----|----|----|----|----|----|----|----|---|---|---|

Supplement: Supplementary file 4 — Supplementary file3 (DOCX 18 KB) [file 432_2022_4096_MOESM4_ESM.pdf]
