# Supplementary material for: Body composition dynamics and impact on clinical outcome in gastric and gastro-esophageal junction cancer patients undergoing perioperative chemotherapy with the FLOT protocol
Source: J Cancer Res Clin Oncol. 2022 Jul 21;149(7):3051–64. doi: 10.1007/s00432-022-04096-w (PMC10314876; doi:10.1007/s00432-022-04096-w)
Supplement: Supplementary file 2 — Supplementary file2 (PDF 32 KB) [file 432_2022_4096_MOESM2_ESM.docx]

**8. Supplementary Material**

**Online Resource 1 CT- and FDG-PET-based body composition parameter analyses**

Cross-Sectional CT images at the level of the third lumbar vertebral body (a-d). Measurement of transverse psoas muscle thickness, psoas muscle area and psoas muscle perimeter prior to pre-operative FLOT (a) and after pre-operative FLOT (b). Measurement of skeletal muscle area prior to pre-operative FLOT (c) and after pre-operative FLOT (d). Brown adipose tissue body composition parameters including volume, Hounsfield, SUVmax and SUVmean were determined bilaterally (blue and red) based on the Region of Interest (ROI) that was drawn in the cervical, supraclavicular and axillary regions until the area of the second rib on CT (e: prior to pre-operative FLOT, f: after pre-operative FLOT) and PET (g: prior to pre-operative FLOT, h: after pre-operative FLOT) imaging. Soft tissues including lymph nodes and muscles were excluded manually from the ROIs.

**Online Resource 2 Baseline characteristics**

| **Baseline Characteristics** | **Number of Patients (%)**  **N=46** |
| --- | --- |
| Median Age  (range: minimum-maximum) | 60.5 years  (46-79) |
| Sex  male  female | 37 (80)  9 (20) |
| ECOG PS at pre-operative FLOT start  0  1  NA | 38 (84)  7 (16)  1 |
| Primary tumor localization  stomach  GEJ | 23 (50)  23 (50) |
| Pre-operative diagnostic laparoscopy  no  yes | 26 (57)  20 (43) |
| Histologic grade  1  2  3 | 1 (2)  11 (24)  34 (74) |
| Histologic subtype  intestinal  diffuse or mixed  NA | 26 (65)  14 (35)  6 |
| HER2 status  negative  positive  NA | 29 (88)  4 (12)  13 |
| cT stage  2  3  4  NA | 16 (37)  21 (49)  6 (14)  3 |
| cN stage  negative  positive  NA | 28 (62)  17 (38)  1 |
| cTNM stage  1  2  3  NA | 12 (27)  18 (41)  14 (32)  2 |
| De-escalation of pre-operative FLOT  no  yes | 39 (85)  7 (15) |
| Number of preoperative FLOT cycles  2  3  4  7 | 1 (2)  2 (4)  42 (92)  1 (2) |
| ypT  0  1  2  3  4 | 5 (11)  13 (28)  8 (17)  15 (33)  5 (11) |
| ypN  0  1  2  3 | 28 (61)  4 (9)  6 (13)  8 (17) |
| ypTNM  0  1  2  3 | 5 (11)  16 (35)  14 (30)  11 (24) |
| Tumor regression grade (Becker)  1a (complete remission)  1b (1-9% residual tumor cells)  2 (10-50% residual tumor cells)  3 (>50% residual tumor cells)  NA | 5 (11)  17 (38)  12 (27)  11 (24)  1 |
| Start of post-operative FLOT  yes  no | 38 (83)  8 (17) |
| ECOG PS at start of post-operative FLOT  0  1  2  NA | 10 (29)  21 (60)  2 (11)  11 |
| De-escalation of post-operative FLOT  yes  no  NA | 25 (68)  12 (32)  1 |
| Number of postoperative FLO(T) cycles  0  1  2  3  4 | 8 (17)  0 (0)  3 (7)  5 (11)  30 (65) |
| ECOG PS at the end of post-operative FLO(T)  0  1  2  NA | 13 (35)  22 (60)  2 (5)  1 |

ECOG PS: Eastern Cooperative Oncology Group performance status, GEJ: gastroesophageal, HER2: human epidermal growth factor receptor 2, NA: not available

**Online Resource 3 Clinical outcome among 46 gastric/GEJ cancer patients undergoing perioperative FLOT**

Online Resource 3a Kaplan-Meier curves (PFS). Online Resource 3b Kaplan-Meier curves (OS). The tick marks on the curve represent censored patients

**Online Resource 4 Cross-study comparison of feasibility and clinical outcome among gastric/GEJ cancer patients undergoing perioperative FLOT**

|  | Salzburg cohort  (n=46) | FLOT4 ([Al-Batran et al 2019](#_ENREF_1))  (n=356 in the  FLOT arm) | RealFLOT ([Giommoni et al 2021](#_ENREF_8))  (n=206) |
| --- | --- | --- | --- |
| Study design | retrospective single-center study | prospective, randomized, controlled trial | observational multicenter study |
| Follow-up (months) | 32 | 43 | 12 |
| PFS (months) | 47 | NA | NA |
| DFS (months) | 45 | 30 | NA |
| OS (months) | not reached | 50 | NA |
| pCR rate | 11% | 16% | 7% |
| At least 4 pre-operative FLOT cycles  (regardless of dose reduction) | 93% | 90% | 90% |
| Full dose 4 pre-operative FLOT cycles | 85% | 81% | 60% |
| Dose-reduction pre-operative FLOT | 15% | 19% | 40% |
| Start of post-operative FLOT | 83%  (38 out of 46) | 60%  (213 out of 356) | 69%  (142 out of 206) |
| 4 post- operative FLOT cycles (regardless of dose reduction) | 65%  (30 out of 46) | 46%  (162 out of 356) | NA |
| Dose-reduction of  post-operative FLOT | 68%  (25 out of 37) | 46%  (97 out of 213) | 54%  (77 out of 142) |

DFS: disease-free survival, pCR: pathologic complete remission, OS: overall survival, PFS: progression-free survival

**Online Resource 5 Interobserver correlation CT-based body composition parameters**

|  | **Pre-FLOT  CT/PET-CT** | **Pre-operative CT/PET-CT** | **1 year^a^ post-operative CT/PET-CT** |
| --- | --- | --- | --- |
| SMI (cm^2^/m^2^) | r=0.978, p<0.001* | r=0.918, p<0.001* | r=0.969, p<0001* |
| TPMT (mm/m^2^) | r=0.829, p<0.001* | r=0.921, p<0.001* | r=0.908, p<0.001* |
| PMA (mm^2^/m^2^) | r=0.962, p<0.001* | r=0.965, p<0.001* | r=0.962, p<0.001* |
| PMP (mm/m^2^) | r=0.903, p<0.001* | r=0.922, p<0.001* | r=0.807, p<0.001* |

*statistically significant (p<0.05)

^a^time window: 10 to 14 months

r: Spearman’s correlation coefficient

SMI: skeletal muscle index, TPMT: transverse psoas muscle thickness, PMA: psoas muscle area, PMP: psoas muscle perimeter

**Online Resource 6 Correlation between baseline anthropometric, CT- and FDG-PET-based body composition parameters**

|  | **Baseline**  **Body weight**  **(kg)** | **Baseline**  **Body mass index**  **(BMI)** | **Baseline**  **Skeletal muscle index**  **(SMI)** |
| --- | --- | --- | --- |
| **Body weight (kg)** | - | - | r=0.557, p<0.001* |
| **BMI (kg/m^2^)** | - | - | r=0.490, p=0.001* |
| **TPMT (mm/m^2^)** | r=0.517, p<0.001* | r=0.463, p=0.002* | r=0.650, p<0.001* |
| **PMA (mm^2^/m^2^)** | r=0.555, p<0.001* | r=0.450, p=0.002* | r=0.663, p<0.001* |
| **PMP (mm/m^2^)** | r=0.395, p=0.008* | r=0.420, p=0.005* | r=0.524, p<0.001* |
| **BAT HU** | r=-0.632, p<0.001* | r=-0.540, p=0.003* | r=-0.002, p=0.99 |
| **BAT SUVmean** | r=-0.144, p=0.47 | r=-0.075, p=0.71 | r=0.265, p=0.18 |
| **BAT volume** | r=0.683, p<0.001* | r=0.708, p<0.001* | r=0.288, p=0.15 |
| **BAT SUVmax** | r=0.373, p=0.05 | r=0.424, p=0.03* | r=0.030, p=0.88 |

*statistically significant (p<0.05)

r: Spearman’s correlation coefficient

BMI: body mass index, TPMT: transverse psoas muscle thickness, PMA: psoas muscle area, PMP: psoas muscle perimeter, BAT: brown adipose tissue, SUV: standard uptake volume, HU: Hounsfield units

**Online Resource 7 Impact of baseline body mass index and skeletal muscle index on the feasibility of perioperative FLOT**

|  | **BMI** |  | **p-value** | **SMI** |  | **p-value** |
| --- | --- | --- | --- | --- | --- | --- |
|  | **No overweight**  **n=19** | **Overweight**  **n=27** |  | **Sarcopenia**  **n=19** | **No sarcopenia**  **n=25** |  |
| Time interval pre-operative FLOT (days) | 43  (14-86) | 44  (33-76) | 0.32 | 45  (41-63) | 42  (14-86) | 0.13 |
| Time interval FLOT start to surgery (days) | 83  (49-120) | 86  (62-119) | 0.65 | 85  (63-119) | 85  (49-120) | 0.32 |
| Number of pre-operative FLOT cycles | 2 cycles: 5%  3 cycles: 5%  4 cycles: 85%  7 cycles: 5% | 2 cycles: 0%  3 cycles: 4%  4 cycles: 96%  7 cycles: 0% | 0.38^a^ | 2 cycles: 0%  3 cycles: 0%  4 cycles: 100%  7 cycles: 0% | 2 cycles: 4%  3 cycles: 4%  4 cycles: 88%  7 cycles: 4% | 0.49^a^ |
| Dose-reduction pre-operative FLOT | 11% | 15% | 0.67^a^ | 11% | 12% | 0.88^a^ |
| Post-operative FLOT start | 95% | 74% | 0.07^a^ | 84% | 80% | 0.72^a^ |
| Number of post-operative FLO(T) cycles | 0 cycles: 5%  1 cycle: 0%  2 cycles: 11%  3 cycles: 21%  4 cycles: 63% | 0 cycles: 26%  1 cycle: 0%  2 cycles: 4%  3 cycles: 4%  4 cycles: 66% | 0.08^a^ | 0 cycles: 16%  1 cycle: 0%  2 cycles: 5%  3 cycles: 11%  4 cycles: 68% | 0 cycles: 20%  1 cycle: 0%  2 cycles: 4%  3 cycles: 12%  4 cycles: 64% | 0.98^a^ |
| Dose-reduction post-operative FLO(T) | 71% | 65% | 0.72^a^ | 60% | 75% | 0.34^a^ |

^a^Chi square test

*statistically significant (p<0.05)
